# Supplementary material for: The interactome of intact mitochondria by cross-linking mass spectrometry provides evidence for coexisting respiratory supercomplexes
Source: Mol Cell Proteomics. 2017 Dec 8;17(2):216–32. doi: 10.1074/mcp.RA117.000470 (PMC5795388; doi:10.1074/mcp.RA117.000470)
Supplement: Supplemental Data [file supp_RA117.000470_133922_0_supp_23978_fzffwf.zip › spectra_annotation/mito_DR_spectra_annotation/176-1-2-1-10-1.pdf]

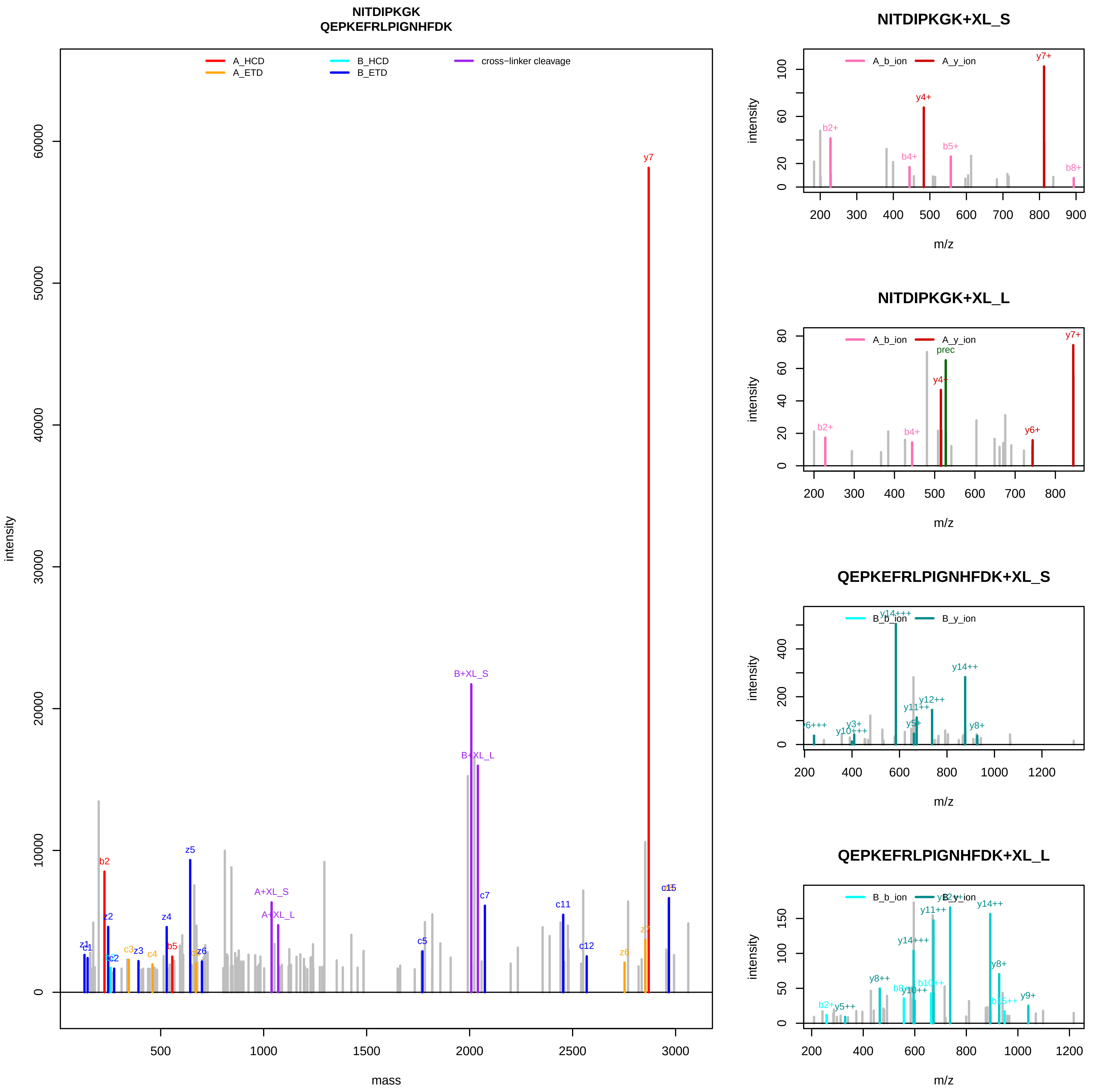
NITDIPKGK
QEPKEFRLPIGNHFDK
A_HCD
A_ETD
B_HCD
B_ETD
cross−linker cleavage
intensity
mass
NITDIPKGK+XL_S
NITDIPKGK+XL_L
QEPKEFRLPIGNHFDK+XL_S
QEPKEFRLPIGNHFDK+XL_L
A_b_ion
A_y_ion
B_b_ion
B_y_ion
m/z
B+XL_S
B+XL_L
A+XL_S
A+XL_L
y7
b2
b5
z1
c1
z2
c2
c3
z3
c4
z4
z5
z6
c5
c7
c11
c12
z6
z7
c15
prec
